# Attenuation of Progressive Hearing Loss in DBA/2J Mice by Reagents that Affect Epigenetic Modifications Is Associated with Up-Regulation of the Zinc Importer Zip4

**DOI:** 10.1371/journal.pone.0124301

**Published:** 2015-04-14

**Authors:** Hideki Mutai, Fuyuki Miya, Masato Fujii, Tatsuhiko Tsunoda, Tatsuo Matsunaga

**Affiliations:** 1 Laboratory of Auditory Disorders, Division of Balance and Hearing Research, National Institute of Sensory Organs, National Tokyo Medical Center, Meguro, Tokyo, Japan; 2 Laboratory for Medical Science Mathematics, RIKEN Center for Integrative Medical Sciences, Yokohama, Kanagawa, Japan; 3 Division of Balance and Hearing Research, National Institute of Sensory Organs, National Tokyo Medical Center, Meguro, Tokyo, Japan; Universitat Pompeu Fabra, SPAIN

## Abstract

Various factors that are important for proper hearing have been identified, including serum levels of zinc. Here we investigated whether epigenetic regulatory pathways, which can be modified by environmental factors, could modulate hearing. RT-PCR detected expression of genes encoding DNA methyltransferase and histone deacetylase (Hdac) in the postnatal as well as adult mouse auditory epithelium. DBA/2J mice, which are a model for progressive hearing loss, were injected subcutaneously with one or a combination of the following reagents: <smallcaps>L</smallcaps>-methionine as a methyl donor, valproic acid as a pan-Hdac inhibitor, and folic acid and vitamin B12 as putative factors involved in age-related hearing loss. The mice were treated from ages 4 to 12 weeks (N ≥ 5), and auditory brainstem response (ABR) thresholds were measured at 8, 16, and 32 kHz. Treatment of the mice with a combination of <smallcaps>L</smallcaps>-methionine and valproic acid (M+V) significantly reduced the increase in the ABR threshold at 32 kHz. Treatment with any of these reagents individually produced no such effect. Microarray analyses detected 299 gene probes that were significantly up- or down-regulated in the cochleae of mice treated with M+V compared with the control vehicle-treated mice. Quantitative RT-PCR confirmed significant up-regulation of a zinc importer gene, *Zip4*, in the cochleae of mice treated with M+V. Immunohistochemistry demonstrated an intense Zip4 signal in cochlear tissues such as the lateral wall, organ of Corti, and spiral ganglion. Finally, mice treated with the *Zip4* inducer (–)-epigallocatechin-3-*O*-gallate showed a significant reduction in the increase of the ABR threshold at 32 kHz and up-regulation of *Zip4* expression in the cochlea. This study suggests that epigenetic regulatory pathways can modify auditory function and that zinc intake in the cochlea via Zip4 mediates maintenance of mammalian hearing.

## Introduction

Hearing loss can be caused by a combination of multiple environmental and genetic factors. A variety of risk factors for progressive hearing loss, including age-related hearing loss, have been identified [1].

Oxidative stress is widely considered a major factor associated with age-related hearing loss [2], whereas other factors are thought to be important for the maintenance of hearing. For instance, proper auditory function requires adequate intake of several nutrients, including zinc [3,4]. A high zinc content has been observed in the guinea pig inner ear [3]. Treatment of normal-hearing CBA mice with a zinc-deficient diet for 8 weeks increases the auditory brainstem response (ABR) threshold at all frequencies measured, an indication of hearing loss [5]. In some elderly adults whose serum levels of zinc are marginally deficient, zinc supplementation can improve sensorineural hearing loss [4]. In addition, some patients with tinnitus have low serum levels of zinc, and their symptoms can be attenuated by zinc supplementation [4,6–8]. In contrast, zinc supplementation for patients with normal serum levels of zinc does not show beneficial effects [9]. Furthermore, the recovery of idiopathic sudden sensorineural hearing loss can be enhanced by zinc supplementation together with steroid administration [10].

Zinc is an essential trace element that modulates a variety of protein functions, such as enzymatic activity, protein structure, and gene expression, and it is important for multiple physiological processes including proper functioning of the pancreas and the immune and nervous systems [11]. The intracellular zinc concentration in mammals is controlled by the ZIP zinc importer (also known as SLC39A) [12] and the ZnT zinc exporter (also known as SLC30A) protein families [13]. Among the 14 members of the ZIP family, ZIP4 plays a crucial role in zinc uptake in the intestines [14]. The green tea flavonoid (–)-epigallocatechin-3-*O*-gallate (EGCG) is one of the reagents that increase zinc intracellular uptake via *Zip4* induction [15]. Other nutrients that may be important for maintaining hearing include vitamin B12 and folate, as lower serum levels of these nutrients are associated with age-related hearing loss [16, 17].

Epigenetic regulatory pathways include DNA methyltransferases (DNMTs), histone deacetylases (HDACs), and non-coding RNAs. These pathways work in combination to change chromatin conformation and alter transcription factor accessibility to promoters, therefore altering gene expression levels (for instance, [18, 19]). The implication of epigenetic mechanisms in hearing has attracted researchers involved in this field [20]. For instance, a search for epigenetic regulatory target genes in the postnatal rat auditory epithelium [21] and the analysis of DNA methylation levels associated with properties of progenitor cells in the mouse auditory epithelium [22] have been documented. Although direct molecular evidence of epigenetic modification in the inner ear has been limited because of the technical challenge of preparing sufficient amounts of cochlear tissue for analysis, reports of several syndromic diseases associated with hearing loss suggest that epigenetic pathways have an important role in proper auditory function. For instance, in patients with facioscapulohumeral muscular dystrophy 1, which includes deafness as an integral clinical phenotype, hypomethylation of the D4Z4 microsatellite repeat on chromosome 4q is linked to the disorder [23]. Mutation of genes involved in epigenetic regulatory pathways such as *DNMT1*, *MECP2*, and *HDAC8* also show multiple symptoms including hearing loss [24–27].

Genetic variants associated with progressive hearing loss have been extensively researched in animal models as well as humans [1]. The DBA/2J (DBA) mouse strain harbors the *Cdh23* ^*753G>A*^ [28] and *Fscn2* ^*326G>A*^ alleles [29], genetic variants associated with progressive hearing loss in mice, and these mice show early-onset, progressive hearing loss—the mice begin to show high-frequency hearing loss at as early as 3 weeks of age and have severe hearing loss by 6 months [28]. Loss of spiral ganglion cells is the main pathological feature during the progression of hearing loss in the DBA mice [30]. Although the progression of hearing loss in these mice is rapid and is, therefore, not entirely comparable to age-related hearing loss in humans, this model is still useful for studying the molecular mechanisms of hearing, as it allows short-term, repeated *in vivo* experiments.

Comprehensive understanding of the molecular pathways involved in hearing may lead to the prevention or treatment of hearing loss. In this study, we investigated whether epigenetic regulatory pathways could modulate the maintenance of mouse auditory function, and we demonstrated that up-regulation of *Zip4* can attenuate progressive hearing loss in DBA mice.

## Materials and Methods

### Animals and treatment conditions

Inbred strains of DBA and FVB/NJ (FVB) mice, which represent a wild-type auditory system, were purchased from Clea Japan (Tokyo, Japan). All animals were housed in plastic cages with metal lids in a room with a 12-h light/dark cycle and 55% humidity at 23°C and had free access to food and water. For *in vivo* treatment with epigenetic modifying reagents, male DBA mice (4 weeks old) were subcutaneously injected once daily for 8 weeks with l-methionine (MET; 500 mg/kg, Wako Pure Chemicals, Osaka, Japan) and/or valproic acid (VPA; 300 mg/kg, Sigma-Aldrich, St Louis, MO), folic acid (FA; 15 mg/kg, Sigma-Aldrich), or vitamin B12 (VB12; 20 mg/kg, Sigma-Aldrich) in 0.1 M sodium bicarbonate (0.1 ml/10 g body weight). The solutions were filtered, aliquoted, and stored at -20°C. A fresh aliquot was thawed weekly and kept at 4°C. In the other experiments, DBA mice were subcutaneously injected three times weekly for 8 weeks with EGCG (95% purity, 5 mg/kg or 10 mg/kg, Mitsui Norin, Shizuoka, Japan) dissolved to 0.05% or 0.1% final percentage in saline. The EGCG solutions were made fresh weekly, filtered, and kept at 4°C. The auditory epithelium was defined here as consisting of the organ of Corti and the spiral limbus [21]. All experimental procedures were approved by the Institutional Animal Care and Use Committee of National Tokyo Medical Center (permit number: 12-animal-02).

### Measurement of ABR

ABR was measured as described [31] with slight modification. In brief, mice were anesthetized by intraperitoneal injection of xylazine-ketamine, and then ABR thresholds were recorded in the left ear of each mouse before and after treatment with serial doses of drugs. Thresholds were recorded with the PowerLab2/20 data acquisition and analysis system (AD Instruments, Castle Hill, Australia). Pure-tone bursts of 8, 16, and 32 kHz (0.2-ms rise/fall time; 1-ms flat segment) and the amplitudes were specified by a real-time processor and programmable attenuator (RP2.1 and PA5, Tucker-Davis Technologies, Alachua, FL). Sound levels were calibrated using a sound level meter (NL32, Rion, Tokyo, Japan). Waveforms of 512 stimuli at each frequency were averaged, and the visual detection threshold was determined with incremental steps of ±5 dB. The thresholds were evaluated as the mean ± standard deviation (s.d.), and statistical significance was determined with a one-way ANOVA and Scheffe's post-hoc test.

### Quantitative real-time PCR (qRT-PCR) and microarray gene expression analysis

RNA extraction, reverse transcription, reverse transcription–PCR (RT-PCR), and qRT-PCR were performed as described [21], with modifications. In brief, total RNA (100 ng) was extracted from the left auditory epithelia of FVB mice (for analyses of *Dnmt*s and *Hdac*s) or from the whole left cochleae of DBA mice (for other genes) at postnatal day 1 (P1), P14, 4 weeks, and 12 weeks with or without drug treatment, or from organs of adult FVB mice. Total RNA was extracted from tissues immediately after dissection using TRIZOL reagent (Life Technologies, Carlsbad, CA), purified using the RNeasy mini kit (Qiagen, Hilden, Germany), and reverse transcribed using SuperScript III (Life Technologies) with random-sequence hexamer primers (TaKaRaBIO, Shiga, Japan). For RT-PCR, 1/100 of the reverse transcription reaction mix was used for each PCR reaction. For qRT-PCR, 1 μl of cDNA was used as the template for each reaction, which was carried out in duplicate, and the reaction products were analyzed with the Prism 7000 system (Applied Biosystems, Foster City, CA) using Power SYBR Green Master Mix (Life Technologies). The standard curves for *Dnmt*s, *Hdac*s, and *Gapdh* were generated using the cDNA generated from ovaries of adult FVB mice at 5 months of age.

For microarray gene expression analysis, the quality of RNA extracted from the whole cochlea of the left inner ear (5–6 mice in each group) was assessed with the Agilent BioAnalyzer 2100 (Agilent Technologies, Santa Clara, CA). The RNA was subjected to a microarray assay using the GeneChip Mouse Genome 430 2.0 Array (Affymetrix, Santa Clara, CA) with 45,101 probes. The expression array data were normalized using the MAS5 algorithm (Affymetrix). To correct for bias between chips, we performed quantile normalization [32] on all array data using the R software. The signal reliability of each probe was determined based on the MAS5 Call algorithm (Affymetrix), and each probe was assigned one of three flags (present, marginal, or absent). To statistically compare microarray data sets, we selected only the probes assigned as “present” for all samples in at least one group for Welch’s *t*-test (two-sided). To validate the microarray data, qRT-PCR was carried out to measure differences in cycle threshold values (ΔCt) between each gene and *Gapdh*. The standard curve for *Zip4* and *Gapdh* was generated using mouse hepatocarcinoma Hepa1-6 cells (The *Zip4/Gapdh* ratio = 1 in Hepa1-6 cells) supplied by the RIKEN BioResource Center (Tsukuba, Japan). Statistical significance was estimated by one-way ANOVA and Scheffe's test (N = 3). The primer sequences for RT-PCR and qRT-PCR are shown in S1 Table.

The dataset from the microarray experiments has been deposited in the National Center for Biotechnology Information Gene Expression Omnibus database (Accession number: GSE62173).

### Gene set enrichment analysis

Gene set enrichment analysis for molecular function and disease was performed using the Ingenuity Pathway Analysis software (Qiagen). The analysis was based on Fisher’s exact test. The data were adjusted for multiple tests using the Benjamini-Hochberg false discovery rate [33].

### Immunohistochemistry

Mice were anesthetized by xylazine-ketamine, perfused with saline, and fixed with 10% formalin. Dissected right inner ears were decalcified for 10–14 days at 4°C and paraffin embedded. Transverse 5-μm-thick sections of the inner ear were rehydrated, pretreated with 10 mM sodium citrate (pH 6.0), and blocked in phosphate-buffered saline with 0.05% Tween-20, 1% bovine serum albumin, and 5% fetal bovine serum. The specimens were incubated with rabbit antiserum against Zip4 (1:50; ab117550, Abcam, Cambridge, UK) and mouse monoclonal antibody against tubulin-βIII (1:500; TuJ1, R&D Systems, Minneapolis, MN) at 4°C overnight. The protein signals were visualized with Alexa 488—or 568–conjugated secondary antibodies (for anti-Zip4 and anti–tubulin-βIII, respectively; Life Technologies) followed by counterstaining with 4',6-diamidino-2-phenylindole (DAPI). Images were captured with confocal microscopy (Bio-Rad Radiance 2100, Bio-Rad, Hercules, CA). Signal specificity of Zip4 staining was determined using intestinal tissue sections from adult FVB mice [34].

## Results

### Expression of *Dnmt*s and *Hdac*s in mouse auditory epithelia

**Fig 1:**
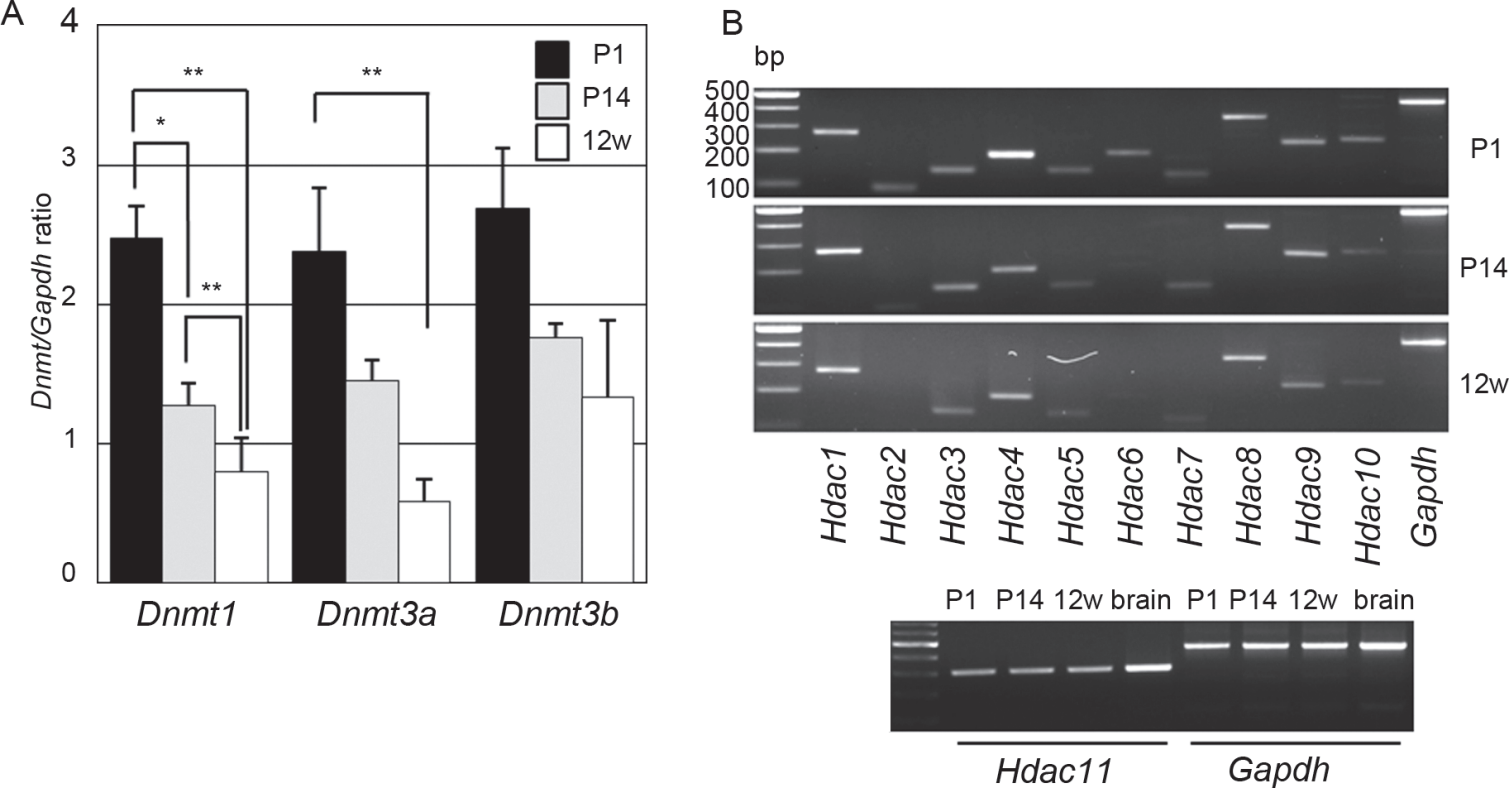
*Dnmt*s and *Hdac*s in the developing mouse auditory epithelium. (A) Expression levels of *Dnmt*s in the auditory epithelia of FVB mice at P1, P14, and 12 weeks (12w). Values shown are the mean ± s.d.; *p < 0.05, **p < 0.01 (N = 3 mice, one-way ANOVA and Scheffe’s test). (B) Expression of *Hdac*s in the developing auditory epithelia of FVB mice as assessed with RT-PCR.

We first examined expression of *Dnmt*s and *Hdac*s in the auditory epithelia of FVB mice with normal hearing. qRT-PCR revealed that the expression levels of all three *Dnmt*s were the highest at postnatal day (P)1 and had decreased by P14, ~4 days after the onset of hearing [35]; the lowest levels were present at 12 weeks (Fig 1A). RT-PCR demonstrated that all 11 *Hdac*s encoded in the mammalian genome were expressed in the mouse cochlea at P1, whereas at 12 weeks, some of the *Hdac*s, such as *Hdac2*, were barely detectable and appeared to be expressed at very low levels (Fig 1B). Expression of *Dnmt*s, *Hdac1*, and *Hdac2* was detectable in various adult organs, with the highest expression levels in spleen among the seven organs examined and relatively high levels in the whole cochlea (S1 Fig), suggesting that epigenetic pathways contribute to cochlear function.

### Treatment of DBA mice with reagents that affect epigenetic modifications

**Fig 2:**
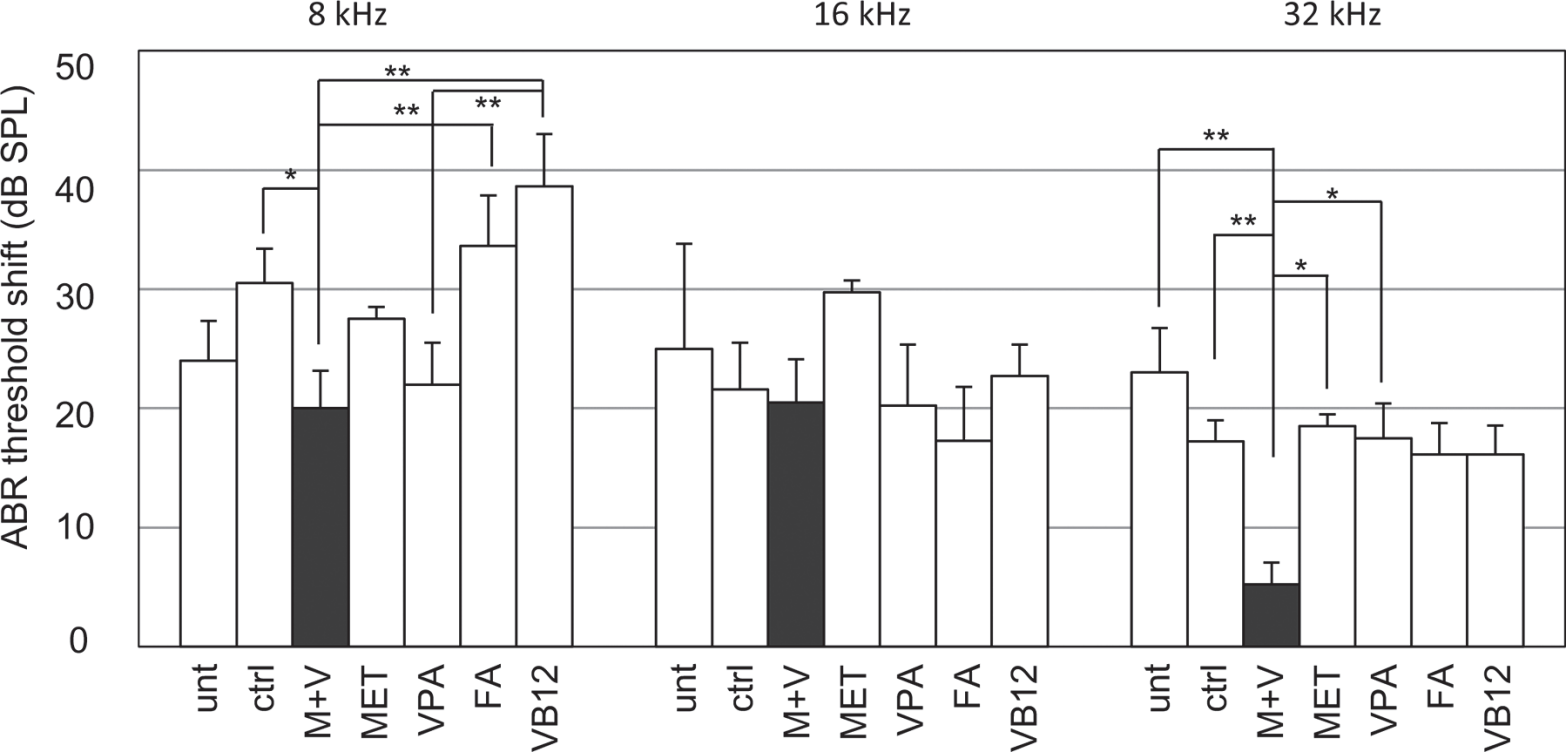
Effects of reagents that affect epigenetic modification on progressive hearing loss in DBA mice. ABR threshold increases at 8, 16, and 32 kHz in DBA mice from 4 to 12 weeks of age that were treated with different reagents. Values shown are the mean ± s.d.; *p < 0.05, **p <0.01 (one-way ANOVA and Scheffe’s test). The numbers of animals in each group were as follows: 5 for untreated (unt), 19 for control (ctrl), 21 for M+V, 10 each for MET and VPA, and 11 each for FA and VB12. SPL, sound pressure level.

To explore the possibility that changes in epigenetic modifications are directly involved in progressive hearing loss, MET, a methyl donor, and VPA, a pan-Hdac inhibitor, were administered individually or together to DBA mice for 8 weeks. We determined ABR levels in each animal at 4 weeks (before treatment) and 12 weeks (8 weeks after treatment) of age (S2 Fig). In untreated mice, ABR thresholds increased by 24 ± 3 dB at 8 kHz, 25 ± 9 dB at 16 kHz, and 23 ± 4 dB at 32 kHz (Fig 2) after 8 weeks. The ABR threshold shifts between untreated and control mice were insignificant at all the frequencies examined. When mice were administered MET together with VPA (M+V), the ABR threshold increased by only 5 ± 2 dB at 32 kHz at 8 weeks, significantly less than in mice treated with VPA (18 ± 3 dB), MET (19 ± 3 dB), or control vehicle (17 ± 2 dB), or in untreated mice. The M+V-treated mice also showed a significantly lower ABR threshold increase at 8 kHz (20 ± 3 dB) at 8 weeks than did those treated with control vehicle (31 ± 3 dB), FA (34 ± 4 dB), or VB12 (39 ± 4 dB). When mice were given VPA, the ABR threshold increased by 22 ± 4 dB at 8 kHz, significantly less than in mice treated with VB12. The MET-treated mice showed hyperactivity, which may be related to the reduced prepulse inhibition of the startle response observed in the animal model of schizophrenia that results from changes in the epigenetic status in the brain after MET treatment [36, 37]. In contrast, the M+V-treated mice recovered from the behavioral abnormalities as reported [37], suggesting that these reagents actually penetrated and facilitated epigenetic modification in the cochlea as well as in the brain.

### Microarray analysis and validation of genes associated with attenuation of progressive hearing loss

To identify possible genes or pathways involved in this reduction in progressive hearing loss induced by reagents that affect epigenetic modifications, total RNA from whole cochleae of mice before and after M+V treatment was subjected to microarray analysis. We hypothesized that genes targeted by epigenetic modification would show differential expression in the M+V-treated cochleae compared with vehicle control as well as untreated cochleae. Fifty-five probes (49 genes) showed a ratio of >1.5-fold for transcript levels in the M+V-treated cochleae compared with the control cochleae, and 244 probes (195 genes) showed a ratio of >1.5-fold for transcript levels in the control cochleae compared with the M+V-treated cochleae (*p* < 0.05, Table 1 and S2 and S3 Tables). Among the genes that were significantly differentially expressed between the M+V-treated and control groups, we selected *Otx1*, *Zip4*, *Calca*, *Pold3*, *Hspa1b*, *Serpinh1*, *Gabra1*, and *Gabrb2* to validate the expression levels by qRT-PCR, based on their known relevance to hearing [38–42]. *Pold3* was also selected because it is involved in DNA replication and repair [43], and we speculated that these functions might have been stimulated in the M+V-treated cochleae. *Gabra1* and *Gabrb2* were considered to be representative genes for neural function, as gene set enrichment analysis based on gene annotations demonstrated that many of the genes down-regulated by M+V were categorized as having a neural function (S4 Table).

**Table 1:** The top 20 significantly up- or down-regulated genes in the M+V-treated cochleae relative to control vehicle-treated cochleae.

Upregulated Genes			Downregulated Genes		
Gene Symbol	Ratio (M+V/ctrl)*	*p* (Welch's t-test)	Gene Symbol	Ratio (M+V/ctrl)	*p* (Welch's t-Test)
***Otx1*** **	2.23	0.037	***Gabra1***	0.12	0.029
***Slc39a4***	2.10	0.011	*Fstl5*	0.16	0.031
*Tbc1d2*	1.98	0.005	***Gabrb2***	0.28	0.016
*Rpl23*	1.96	0.012	*Kcnd2*	0.28	0.022
*Tmem170*	1.94	0.024	*Slitrk4*	0.28	0.024
***Calca***	1.89	0.005	*Gap43*	0.29	0.039
*Sumf1*	1.86	0.049	*Slc25a31*	0.37	0.034
***Pold3***	1.84	0.004	*Cacnb4*	0.41	0.013
*9030619P08Rik*	1.80	0.029	*6330527O06Rik*	0.42	0.046
*Dnahc7a*	1.76	0.023	*B3galt2*	0.43	0.041
*3300002P09Rik*	1.73	0.012	*Mageb16*	0.45	0.027
***Hspa1b***	1.72	0.007	*Rbl1*	0.46	0.022
*Aox3*	1.72	0.046	*Scn1a*	0.46	0.033
*Erbb2ip*	1.69	0.0005	*Cadm2*	0.47	0.015
*Adig*	1.68	0.047	*Pcdh8*	0.48	0.008
***Serpinh1***	1.66	0.050	*A530030E21Rik*	0.48	0.013
*Ntf5*	1.66	0.019	*Clip1*	0.48	0.033
*Fn3krp*	1.65	0.017	*Rictor*	0.49	0.003
*Klf6*	1.65	0.020	*Hs6st2*	0.49	0.040
*AU019752*	1.63	0.027	*Tbx15*	0.49	0.0005

*M+V, l-methionine and valproic acid; ctrl, vehicle control.

**Genes in bold were validated by qRT-PCR.

Among the genes analyzed by qRT-PCR, *Zip4* and *Hspa1b* showed significantly higher expression levels in the M+V-treated cochleae than in the control cochleae (S3 Fig), validating the microarray analysis. *Zip4* was selected for further investigation because it was up-regulated in the M+V-treated cochleae, and because its expression seemed to be inversely associated with ABR threshold values.

**Fig 3:**
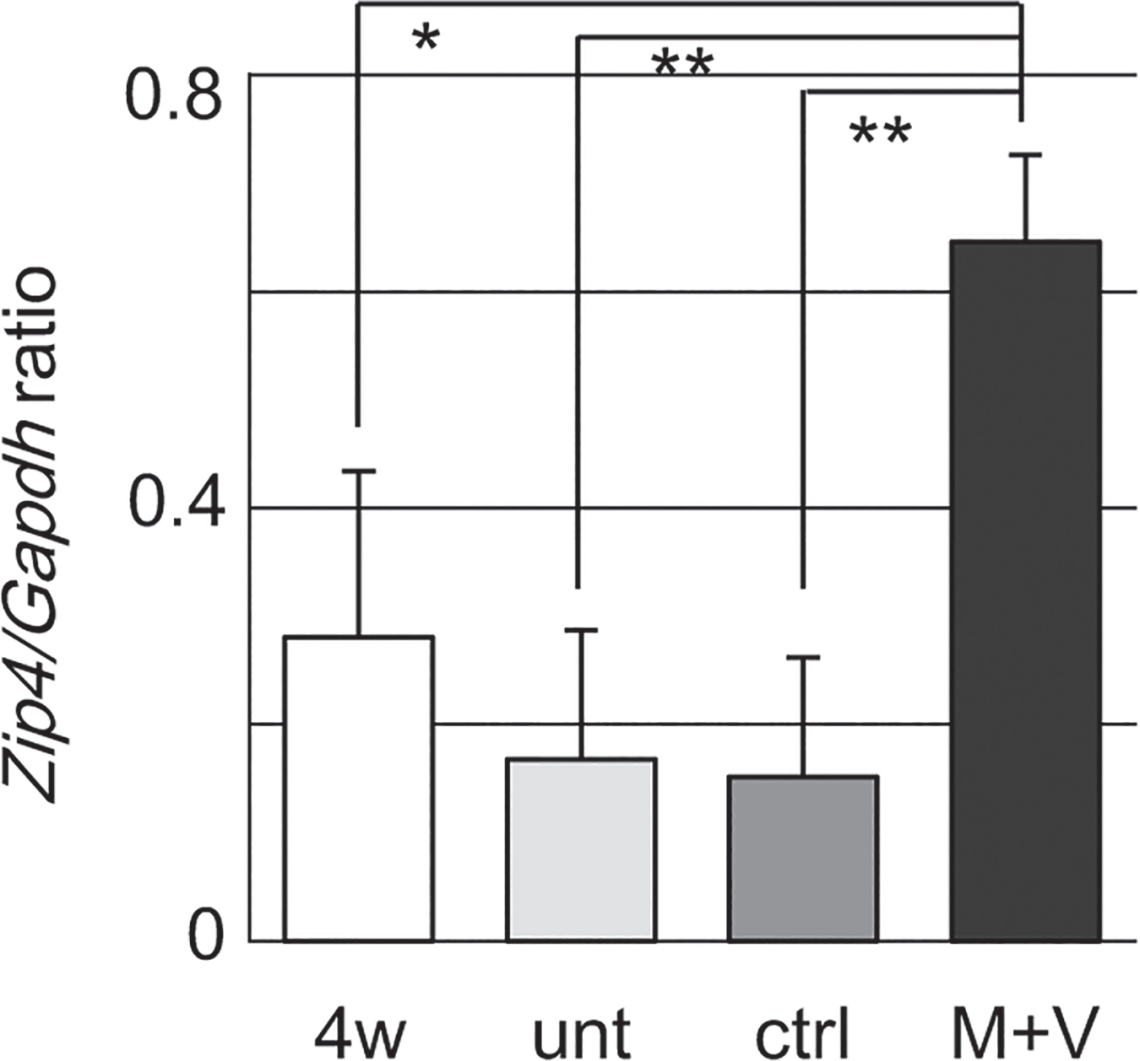
Expression of *Zip4* in the cochlea of DBA mice. *Zip4/Gapdh* ratios in the left whole cochleae of DBA mice at 4 weeks of age (4w) and in untreated cochleae (unt) and cochleae treated with vehicle control (ctrl) or with M+V from DBA mice at 12 weeks of age. N = 3 mice. Values shown are the mean ± s.d.; *p < 0.05, **p <0.01 (one-way ANOVA and Scheffe’s test).

**Fig 4:**
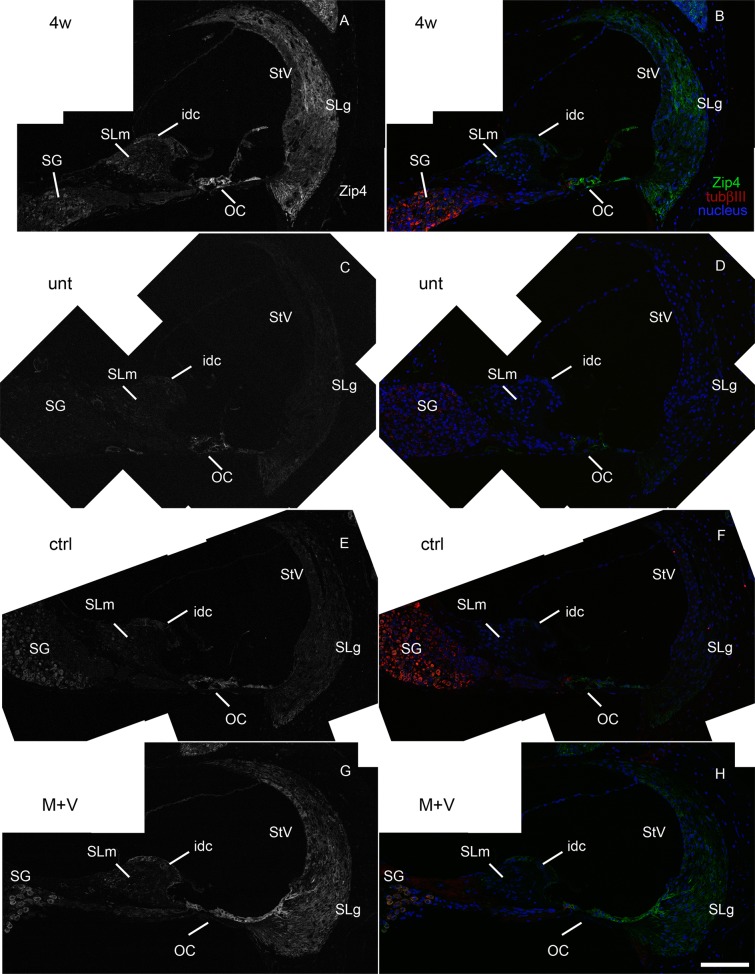
Distribution of Zip4 in the cochlea of DBA mice. Cochlear specimens from 4-week-old (4w; A, B) and untreated (unt) 12-week-old (C, D) mice and from 12-week-old mice treated with vehicle control (ctrl; E, F) or with M+V (G, H) were incubated with rabbit antiserum against Zip4 (green) and monoclonal antibody against tubulin βIII (red). A, C, E, and G are dark-field images of B, D, F, and H, respectively. Scale bar = 100 μm. idc, interdental cell region; OC, organ of Corti; SG, spiral ganglion; SLg, spiral ligament; SLm, spiral limbus; StV, stria vascularis.

**Fig 5:**
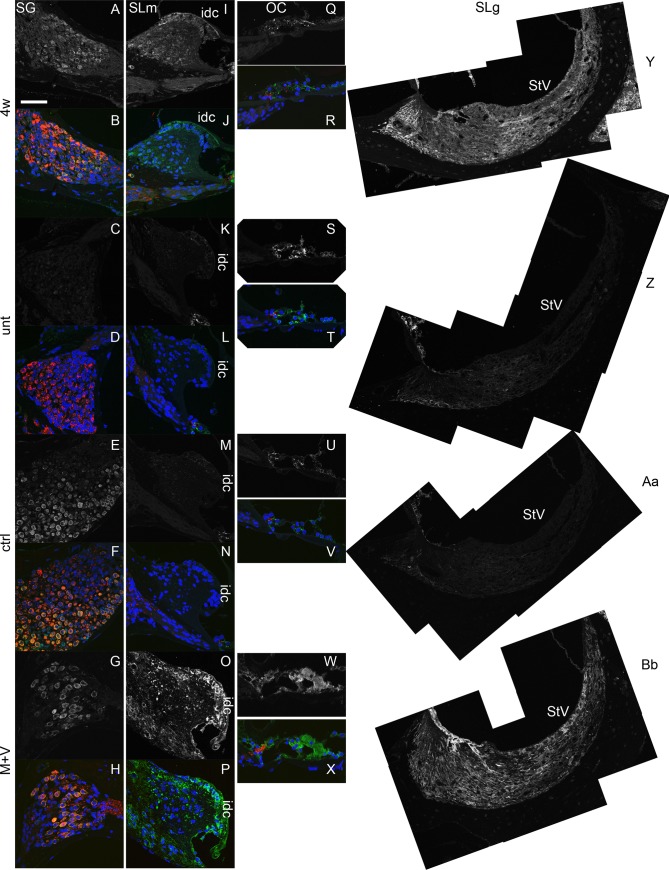
Magnified images of Zip4 expression in the cochlea of DBA mice. Magnified images of spiral ganglion (SG; A-H), spiral limbus (SLm; I-P), organ of Corti (OC; Q-X), and spiral ligament (SLg; Y-Bb) region after incubation with rabbit antiserum against Zip4 (green and signal in dark-field images) and monoclonal antibody against tubulin βIII (red). Cochlear specimens were from 4-week-old (4w; A, B, I, J, Q, R, Y) and untreated (unt) 12-week-old (C, D, K, L, S, T, Z) mice and from 12-week-old mice treated with vehicle control (ctrl; E, F, M, N, U, V, Aa) or M+V (G, H, O, P, W, X, Bb). Scale bar = 50 μm.

Additional qRT-PCR analyses demonstrated that the *Zip4*/*Gapdh* ratio increased 3- to 4-fold from the untreated (0.17 ± 0.12) and the vehicle control cochleae (0.15 ± 0.11) to the M+V-treated cochleae (0.65 ± 0.08) at 12 weeks (Fig 3). The M+V-treated cochleae also showed a significantly higher *Zip4/Gapdh* ratio than the cochleae at 4 weeks (before drug treatment, 0.28 ± 0.15). Zip4 expression, which is predominantly in the adult mouse intestine [34] (S4 Fig), was detectable by immunohistochemistry in several cochlear tissues, including the spiral ganglion; the organ of Corti, which consists of sensory hair cells and supporting cells; and surrounding tissues such as the spiral limbus, which includes interdental cells at its border along the endolymph, and the spiral ligament, which harbors several types of fibrocytes in 4-week-old mice [44] (Figs 4A and 4B and 5A, 5B, 5I, 5J, 5Q, 5R and 5Y). The signal was relatively intense in the organ of Corti and spiral ligament, especially in the area beneath the stria vascularis and inferior to the basilar membrane of the organ of Corti. In the untreated mice at 12 weeks, the Zip4 signal remained detectable in the organ of Corti and the inferior region of the spiral ligament but was less intense in the other regions (Figs 4C and 4D and 5C, 5D, 5K, 5L, 5S, 5T and 5Z). The Zip4 signal intensities in the control mice at 12 weeks were similar to the untreated mice, but the spiral ganglion cells had a stronger signal (Figs 4E and 4F and 5E, 5F, 5M, 5N, 5U, 5V and 5Aa). In the M+V-treated mice at 12 weeks, the Zip4 signal was more intense throughout the cochlear tissue than in the untreated or control mice, especially in the spiral ganglion, spiral limbus, organ of Corti, stria vascularis, and spiral ligament (Figs 4G and 4H and 5G, 5H, 5O, 5P, 5W, 5X and 5Bb). No apparent morphological alterations, such as a decrease in the number of spiral ganglion cells, were observed.

### Treatment of DBA mice with the *Zip4* inducer EGCG

**Fig 6:**
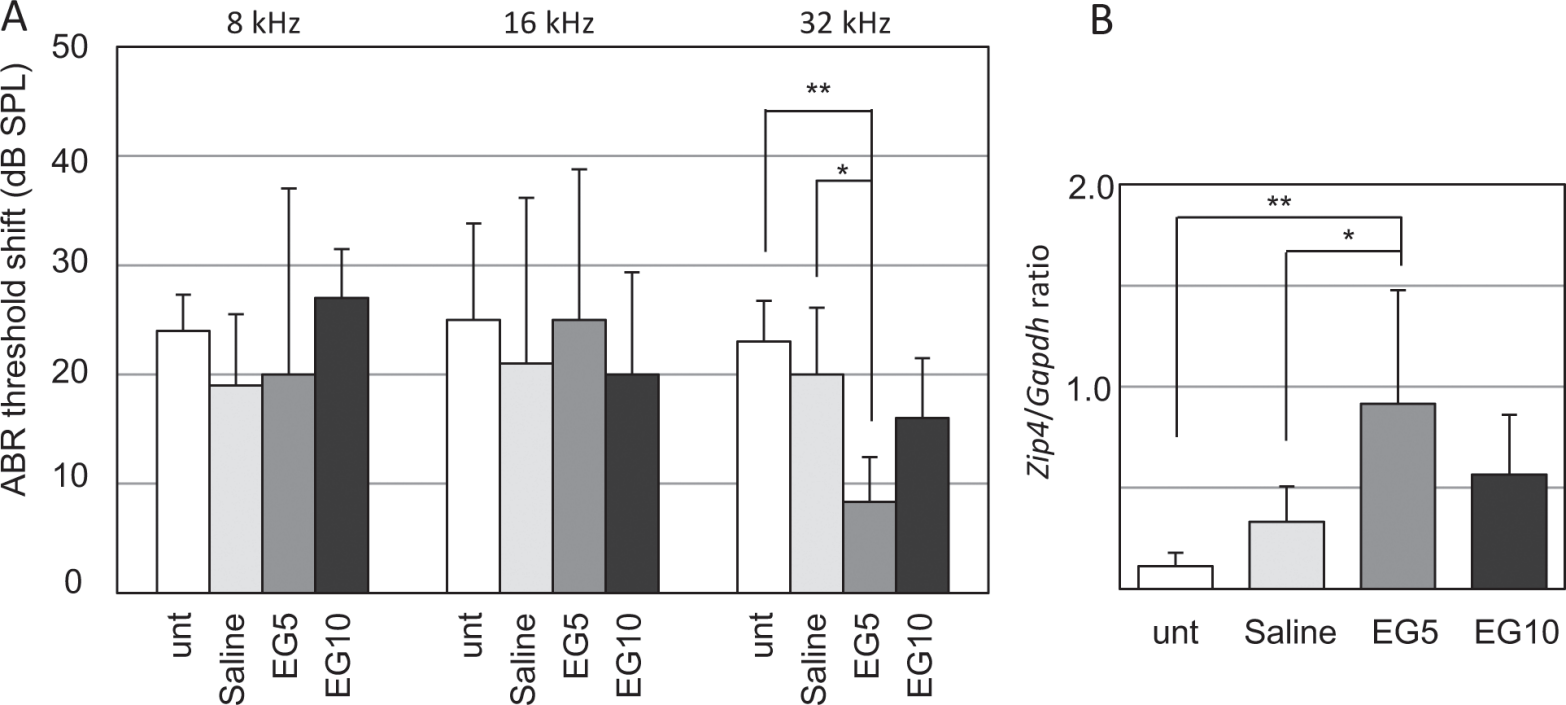
Effect of EGCG treatment on progressive hearing loss in DBA mice. (A) ABR threshold increases at 8, 16, and 32 kHz in DBA mice treated with different doses of EGCG; N = 5 untreated (unt) mice, 5 saline-treated mice, 6 EG5 mice, and 5 EG10 mice. (B) Expression levels of *Zip4* in the whole cochleae from mice treated with EGCG and from untreated and saline-treated mice. N = 3 mice per group. Values shown are the mean ± s.d.; *p < 0.05, **p <0.01 (one-way ANOVA and Scheffe’s test).

To examine whether up-regulation of *Zip4* could affect progressive hearing loss in DBA mice, we administered EGCG to DBA mice from 4 to 12 weeks of age. In mice treated with EGCG at 5 mg/kg (EG5), the ABR threshold increased by 8 ± 4 dB at 32 kHz, significantly less than untreated (23 ± 4 dB) and saline-treated (20 ± 6 dB) mice, or mice treated with EGCG at 10 mg/kg (16 ± 5 dB, EG10) (Fig 6A and S5 Fig). The ABR threshold increase in the EG10 mice was not significantly different than in untreated or saline-treated mice. In addition, qRT-PCR showed that the *Zip4*/*Gapdh* ratio was significantly higher in the EG5-treated cochleae (0.91 ± 0.56) than in the untreated (0.11 ± 0.10) or saline-treated (0.33 ± 0.18) cochleae (Fig 6B). The *Zip4*/*Gapdh* ratio in the EG10-treated cochleae (0.56 ± 0.30) did not show statistical significance compared with the other groups of cochleae. Expression levels of *Otx1*, *Hspa1b*, *Gabra1*, and *Gabrb2* did not show significant differences after EGCG treatment (S6 Fig).

## Discussion

This study strongly suggests that epigenetic regulatory pathways can alter auditory function and identified Zip4 as a likely participant in maintaining hearing in DBA mice.

### Modulation of zinc homeostasis

Our results support previous studies in elderly adults with hearing loss [4], patients with tinnitus [6–8] or idiopathic sudden sensorineural hearing loss [9], and animal models [3,5], all of which have suggested that zinc uptake into cochlear cells and the regulation of molecular pathways underlying the uptake of this mineral are important for maintaining mammalian auditory function. The presence of Zip4 in many cochlear tissues suggests that zinc participates in multiple cellular processes that are important for proper cochlear function. It should be noted that Zip4 expression in some cochlear tissues, such as the organ of Corti and a subregion of the spiral ligament, was detectable in untreated, control, and M+V-treated mice, whereas expression in the spiral limbus and most of the spiral ligament was undetectable in the untreated and control mice but was intense in the M+V-treated mice. Therefore, M+V-induced up-regulation of *Zip4* in the whole cochlea, as detected by microarray analysis and by qRT-PCR, suggests a combination of two effects: enhanced expression in the cochlear cells that express *Zip4* endogenously and ectopic expression of *Zip4* in the cochlear cells with undetectable expression in the uninduced state. Differences in the Zip4 signal intensity in the spiral ganglion cells among untreated, control, and M+V treated mice might be related to the injection of the solvent and not to the attenuation of progressive hearing loss. Intriguingly, cochlear tissues are rich in several enzymes that are affected by intracellular zinc, such as Cu/Zn superoxide dismutase and carbonic anhydrase [45,46]. Our findings appear to contradict the observation that a zinc-deficient diet does not affect hearing in rats [47]. This discrepancy between our findings might be explained by differences in experimental procedures, most obviously in the method of measuring hearing (click sound stimulation in Hoeve *et al*. [47] and tone burst stimulation in this study) or by species-specific zinc requirements in hearing.

Although gene set enrichment analysis demonstrated that the expression of multiple genes related to neural function was changed by M+V (S4 Table), additional and more-extensive analyses are necessary to determine if these genes contribute to the maintenance of hearing in DBA mice. In addition, this study does not exclude the possibility that other candidate genes, such as *Hspa1b* [40], play a role in the improvement in hearing in the M+V-treated mice. A previous study found that multiple genes involved in the mitochondrial respiratory chain or apoptotic pathways show age-related changes in expression in the cochleae of DBA mice [48], but changes in these genes were scarcely detected in our study (S4 Table). This discrepancy is probably due to the fact that this study used younger mice (under 12 weeks old), in which the cochlear cells showed no apparent morphological degeneration, suggesting that they had not accumulated sufficient oxidative damage to activate apoptotic pathways.

### MET and VPA as reagents that affect epigenetic modification in mammalian cochleae

Detection of *Dnmt* and *Hdac* expression in functionally mature mouse auditory epithelium suggests that epigenetic regulatory pathways are involved in auditory function. The significant prevention of progressive hearing loss by M+V, but not MET or VPA alone, strongly suggests that epigenetic modification through multiple pathways synergistically alters the transcriptional profile in cochlear cells and leads to prevention of hearing decline. The anti-oxidative effect of l-methionine, which can prevent cisplatin-induced ototoxic effects to the same extent as d-methionine [49], was insufficient in this study to prevent the decline of hearing in DBA mice. It is plausible that excessive l-methionine would have resulted in increased amounts of *S*-adenosylmethionine, a direct methyl donor to DNA as well as histones, and thus modification of gene expression. Although Hdac inhibitors have been reported to prevent aminoglycoside-induced hair cell death *in vitro* [50] and cisplatin-induced hearing loss in guinea pigs [51], VPA alone was insufficient to prevent hearing loss in this study. Further experiments are necessary to reveal how these M+V-induced changes in the epigenetic profile ultimately lead to modulation of *Zip4* expression and attenuation of progressive hearing loss. Although indirect effects on hearing related to changes in the physiological or metabolic status of other organs cannot be excluded, the relatively abundant expression levels of *Dnmt*s, *Hdac1*, and *Hdac2* in the cochlea support the idea that attenuation of progressive hearing loss by *in vivo* treatment with M+V is associated with direct modification of epigenetic status in cochlear cells.

Low serum levels of both FA and VB12 are associated with age-related hearing loss [16,52], but we did not observe a preventative effect of either of these nutrients on progressive hearing loss in DBA mice; in fact, VB12 treatment led to deteriorated hearing at 8 kHz. The reasons for this difference and for the deterioration are still unknown.

### Prevention of progressive hearing loss in DBA mice by EGCG

The comparable reduction in ABR threshold increase at 32 kHz in the presence of EGCG-induced *Zip4* expression in the cochleae or M+V treatment suggests that Zip4 induction is a major component in the attenuation of hearing loss in DBA mice. Although EGCG has multiple biological activities such as anti-angiogenic and anti-tumor activity [53], we have detected only *Zip4* induction by EGCG treatment, consistent with the effects of M+V treatment, which showed similar attenuation of progressive hearing loss in DBA mice. An alternative mechanism of EGCG treatment—other than *Zip4* induction—that might have attenuated progressive hearing loss in DBA mice is its antioxidant effect. However, because treatment with MET alone failed to attenuate progressive hearing loss in our study and because stimulation of anti-oxidative activities by EGCG has been shown only in aged (not young) rat brains [54], we do not favor the possibility that EGCG ameliorated the loss of hearing in these young DBA mice through anti-oxidative activities when they had not shown any notable tissue damage. Comprehensive understanding of the molecular pathways affected by M+V and EGCG that lead to the induction of *Zip4* and other as-yet-unidentified target genes may help in the development of preventive therapies for hearing loss.

The significant attenuation of progressive hearing loss in DBA mice treated with EGCG at 5 mg/kg, but not with 10 mg/kg, may be indicative of the effective range of EGCG doses. This finding agrees with the previous observation that dietary EGCG at 250 mg/kg does not attenuate hearing loss in C57BL/6 mice, another model of progressive hearing loss [55].

In conclusion, this study suggests that epigenetic regulatory pathways can modify auditory function and that zinc intake in the cochlea through Zip4 mediates the maintenance of mammalian hearing. Manipulation of the expression of genes to enhance zinc intake in the cochlea should be explored as an alternative therapeutic strategy for preventing progressive hearing loss.

## Supporting Information

S1 Fig Expression levels of *Dnmt*s and *Hdac*s in adult mouse organs. Values shown are the mean ± s.d.; *p < 0.05, **p < 0.01 (one-way ANOVA and Scheffe’s test).
(PDF)

S2 Fig ABR threshold of DBA mice treated with epigenetic-modifying reagents. ABR thresholds were recorded in the left ear of each mouse before (4 weeks old, black) and after treatment (12 weeks old, orange) with the noted drugs. Values are shown as the mean ± s.d.
(PDF)

S3 Fig Expression of candidate genes in the whole cochleae of DBA mice treated with M+V. Expression levels were assessed by qRT-PCR and quantified by the ΔCt method for each gene and compared with *Gapdh*. *Otx1*, *Zip4*, *Calca*, *Hspa1b*, *Pold3*, and *Serpinh1* were selected from the list of genes with significantly higher transcripts and *Gabra1* and *Gabrb2* from the list with significantly lower transcripts in M+V-treated cochleae relative to control cochleae. Data are shown as the mean ± s.d.; *p < 0.05; **p < 0.01 (N = 3 mice, one-way ANOVA and Scheffe’s test).
(PDF)

S4 Fig Distribution of Zip4 in the adult mouse intestine. Intestinal specimens from adult FVB mice were incubated with (A, B) or without (C, D) rabbit antiserum against Zip4 (green). A and C are dark-field images of B and D. Scale bar = 50 μm.
(TIF)

S5 Fig ABR threshold of DBA mice treated with EGCG. ABR thresholds were recorded in the left ear of each mouse before (4 weeks old, black) and after (12 weeks old, orange) treatment with 5 mg/kg EGCG (EG5), 10 mg/kg EGCG (EG10), or saline control. Values are shown as the mean ± s.d.
(PDF)

S6 Fig Expression of candidate genes in the whole cochleae of DBA mice treated with EGCG. Expression levels were assessed by qRT-PCR and quantified by the ΔCt method for each gene and compared with *Gapdh*. *Otx1*, *Hspa1b*, *Gabra1*, and *Gabrb2* were analyzed. Data are shown as the mean ± s.d. (N = 3 mice). No statistical difference was detected by one-way ANOVA.
(PDF)

S1 Table Primers used in this study. (XLSX)

S2 Table Genes with significantly higher levels of transcripts in M+V-treated cochleae than in control cochleae. (XLSX)

S3 Table Genes with significantly lower levels of transcripts in M+V-treated cochleae than in control cochleae. (XLSX)

S4 Table Gene set enrichment analysis for the genes up- or down-regulated by M+V treatment. (XLSX)
