# Supplementary material for: Attenuation of Progressive Hearing Loss in DBA/2J Mice by Reagents that Affect Epigenetic Modifications Is Associated with Up-Regulation of the Zinc Importer Zip4
Source: PLoS One. 2015 Apr 14;10(4):e0124301. doi: 10.1371/journal.pone.0124301 (PMC4397065; doi:10.1371/journal.pone.0124301)
Supplement: S1 Fig — Values shown are the mean ± s.d.; *p < 0.05, **p < 0.01 (one-way ANOVA and Scheffe’s test). (PDF) [file pone.0124301.s001.pdf]

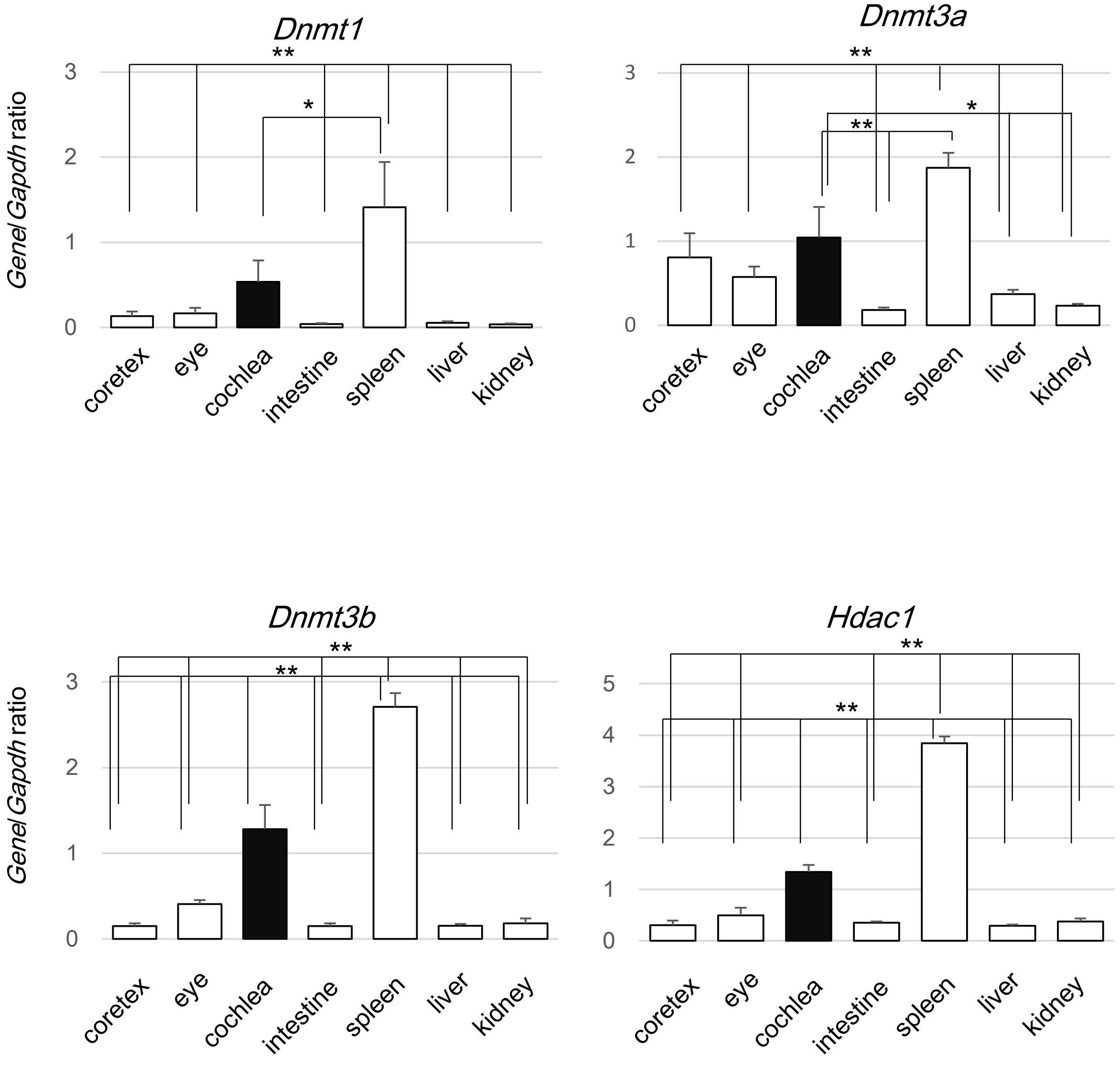

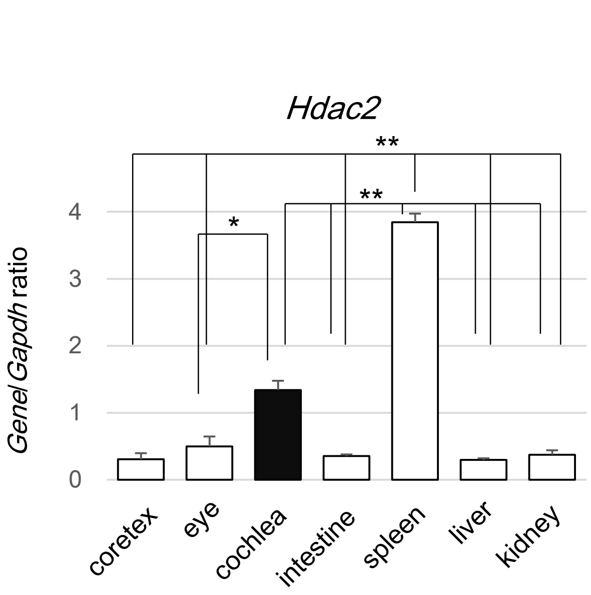

S1_Fig. Expression levels of *Dnmt*s and *Hdac*s in adult mouse organs. Values shown are the mean ± s.d.; *p < 0.05, **p < 0.01 (one-way ANOVA and Scheffe’s test).
