# Supplementary material for: Attenuation of Progressive Hearing Loss in DBA/2J Mice by Reagents that Affect Epigenetic Modifications Is Associated with Up-Regulation of the Zinc Importer Zip4
Source: PLoS One. 2015 Apr 14;10(4):e0124301. doi: 10.1371/journal.pone.0124301 (PMC4397065; doi:10.1371/journal.pone.0124301)
Supplement: S3 Fig — Expression levels were assessed by qRT-PCR and quantified by the ΔCt method for each gene and compared with Gapdh. Otx1, Zip4, Calca, Hspa1b, Pold3, and Serpinh1 were selected from the list of genes with significantly higher transcripts and Gabra1 and Gabrb2 from the list with significantly lower transcripts in M+V-treated cochleae relative to control cochleae. Data are shown as the mean ± s.d.; *p < 0.05; **p < 0.01 (N = 3 mice, one-way ANOVA and Scheffe’s test). (PDF) [file pone.0124301.s003.pdf]

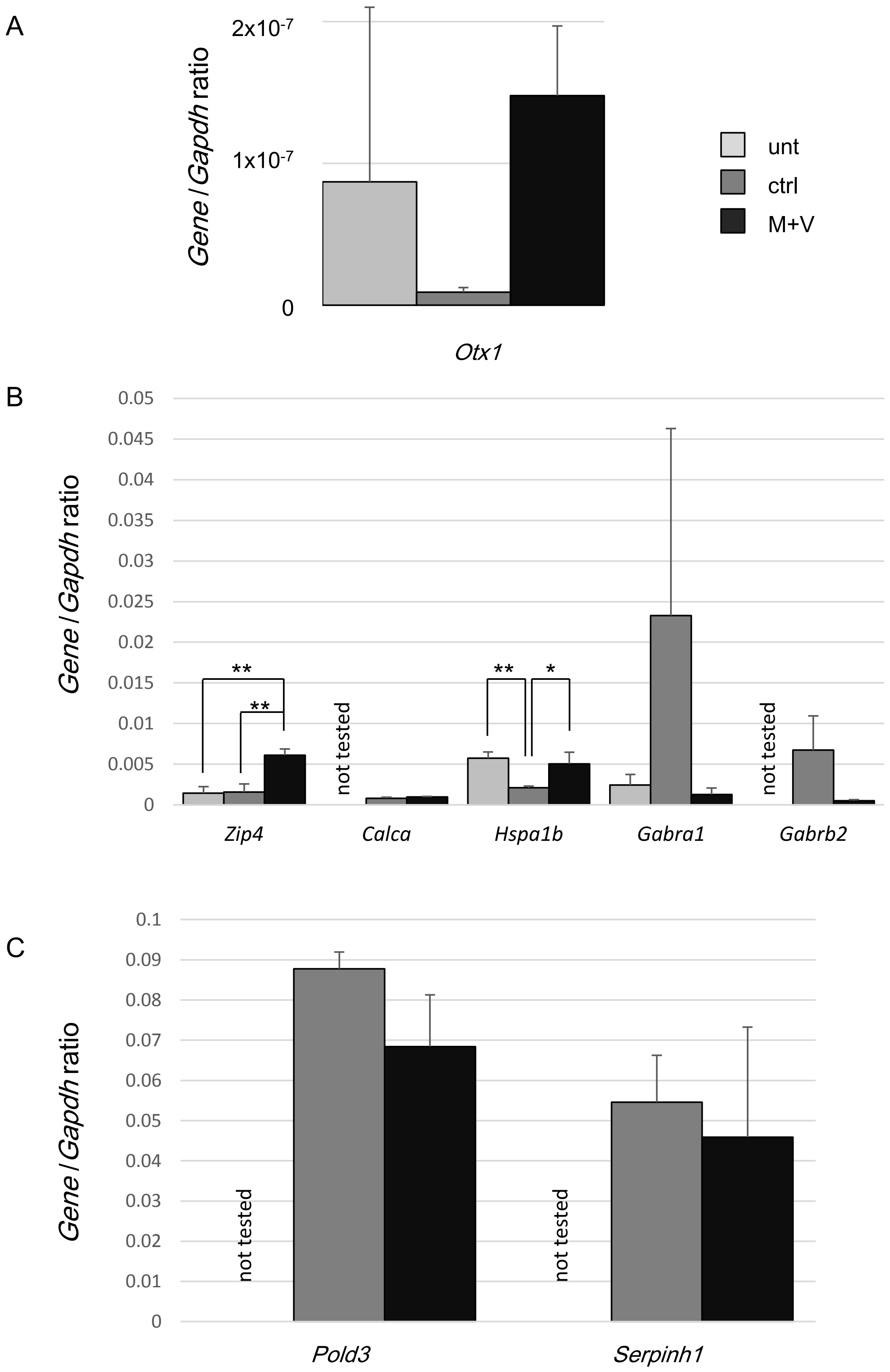

S3_Fig. Expression of candidate genes in the whole cochleae of DBA mice treated with M+V. Expression levels were assessed by qRT-PCR and quantified by the ΔCt method for each gene and compared with *Gapdh*. *Otx1, Zip4, Calca, Hspa1b, Pold3*, and *Serpinh1* were selected from the list of genes with significantly higher transcripts and *Gabra1* and *Gabrb2* from the list with significantly lower transcripts in M+V-treated cochleae relative to control cochleae. Data are shown as the mean ± s.d.; *$p < 0.05$; **$p < 0.01$ (N = 3 mice, one-way ANOVA and Scheffe's test).
