# Supplementary figures and images for: Attenuation of Progressive Hearing Loss in DBA/2J Mice by Reagents that Affect Epigenetic Modifications Is Associated with Up-Regulation of the Zinc Importer Zip4
Source: PLoS One. 2015 Apr 14;10(4):e0124301. doi: 10.1371/journal.pone.0124301 (PMC4397065; doi:10.1371/journal.pone.0124301)

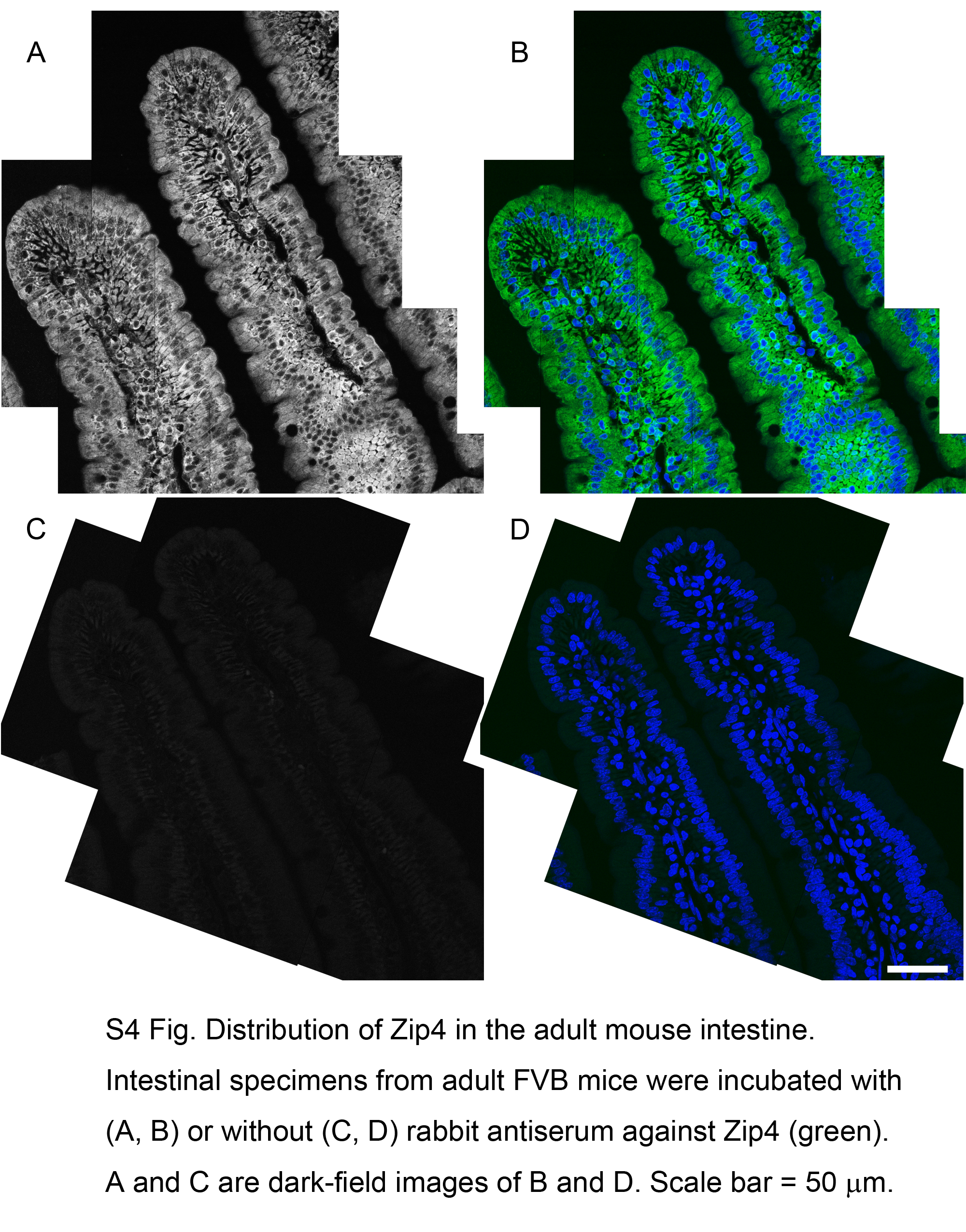

Supplement: S4 Fig — Intestinal specimens from adult FVB mice were incubated with (A, B) or without (C, D) rabbit antiserum against Zip4 (green). A and C are dark-field images of B and D. Scale bar = 50 μm. (TIF) [file pone.0124301.s004.tif]
