# Supplementary material for: Attenuation of Progressive Hearing Loss in DBA/2J Mice by Reagents that Affect Epigenetic Modifications Is Associated with Up-Regulation of the Zinc Importer Zip4
Source: PLoS One. 2015 Apr 14;10(4):e0124301. doi: 10.1371/journal.pone.0124301 (PMC4397065; doi:10.1371/journal.pone.0124301)
Supplement: S5 Fig — ABR thresholds were recorded in the left ear of each mouse before (4 weeks old, black) and after (12 weeks old, orange) treatment with 5 mg/kg EGCG (EG5), 10 mg/kg EGCG (EG10), or saline control. Values are shown as the mean ± s.d. (PDF) [file pone.0124301.s005.pdf]

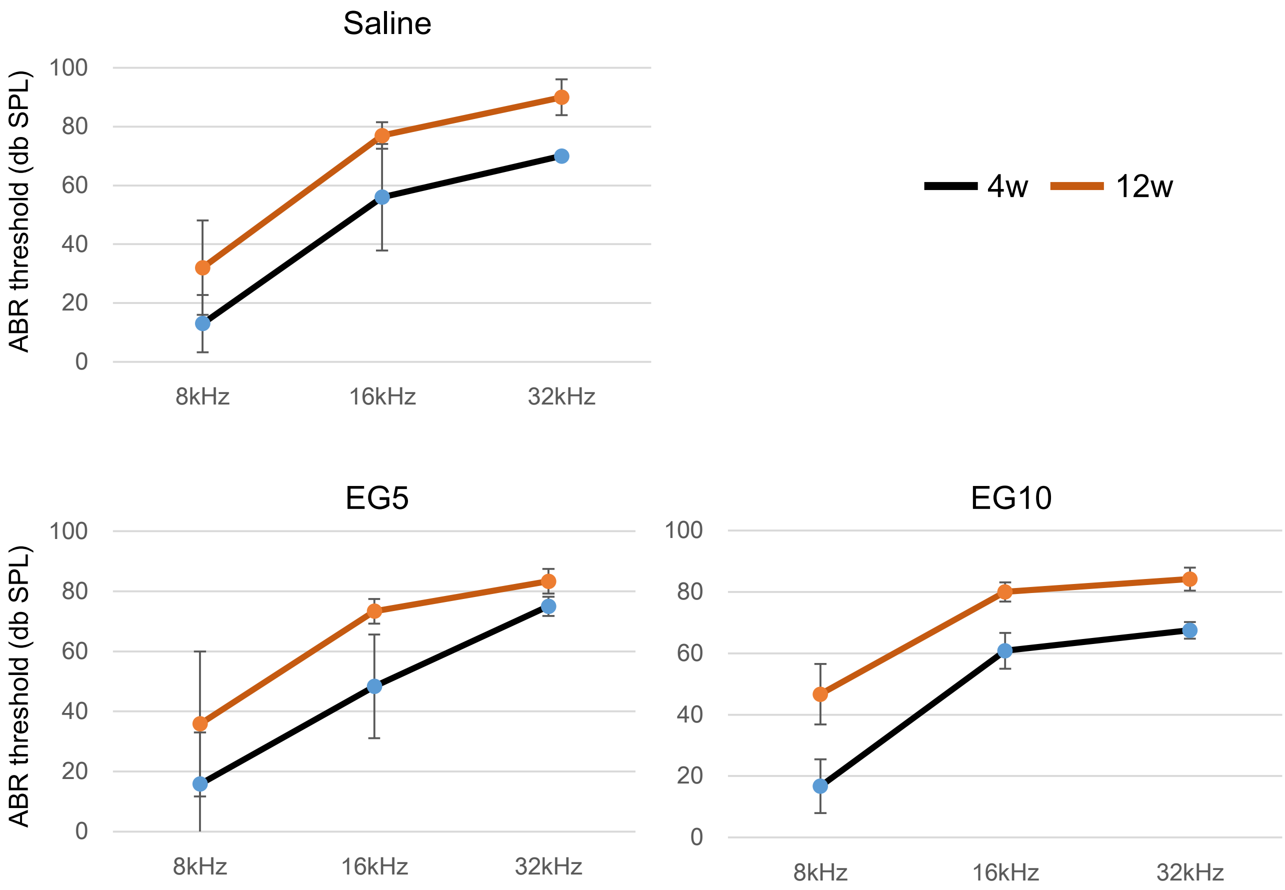

S5_Fig. ABR threshold of DBA mice treated with EGCG. ABR thresholds were recorded in the left ear of each mouse before (4 weeks old, black) and after (12 weeks old, orange) treatment with 5 mg/kg EGCG (EG5), 10 mg/kg EGCG (EG10), or saline control. Values are shown as the mean ± s.d.
