# Supplementary material for: Attenuation of Progressive Hearing Loss in DBA/2J Mice by Reagents that Affect Epigenetic Modifications Is Associated with Up-Regulation of the Zinc Importer Zip4
Source: PLoS One. 2015 Apr 14;10(4):e0124301. doi: 10.1371/journal.pone.0124301 (PMC4397065; doi:10.1371/journal.pone.0124301)
Supplement: S6 Fig — Expression levels were assessed by qRT-PCR and quantified by the ΔCt method for each gene and compared with Gapdh. Otx1, Hspa1b, Gabra1, and Gabrb2 were analyzed. Data are shown as the mean ± s.d. (N = 3 mice). No statistical difference was detected by one-way ANOVA. (PDF) [file pone.0124301.s006.pdf]

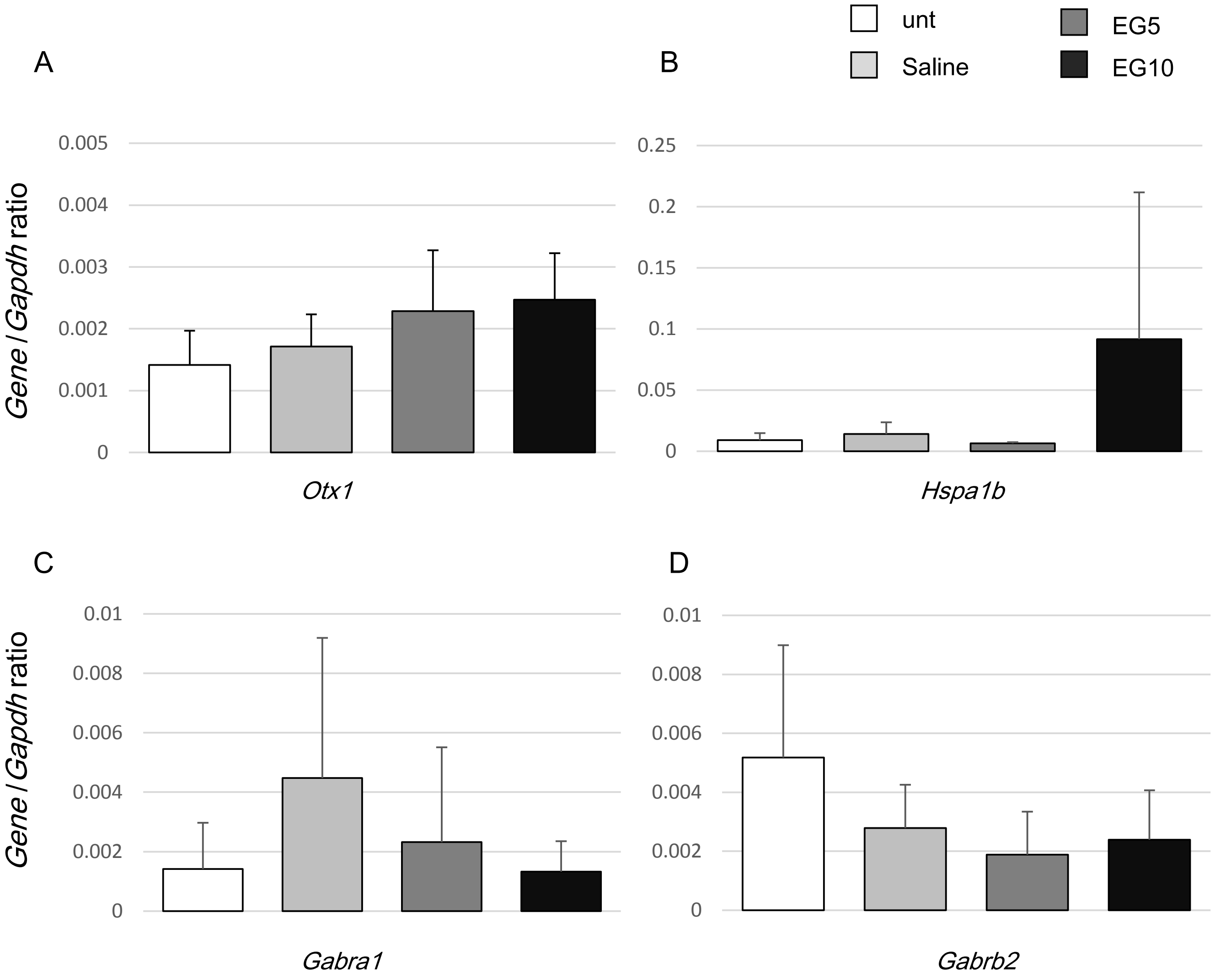

S6_Fig. Expression of candidate genes in the whole cochleae of DBA mice treated with EGCG. Expression levels were assessed by qRT-PCR and quantified by the ΔCt method for each gene and compared with *Gapdh*. *Otx1, Hspa1b, Gabra1*, and *Gabrb2* were analyzed. Data are shown as the mean ± s.d. (N = 3 mice). No statistical difference was detected by one-way ANOVA.
